# Wen(n) die Katze kratzt … Pruriginöse Papeln und Noduli nach Katzenkratzer

**DOI:** 10.1007/s00105-022-05055-9

**Published:** 2022-09-06

**Authors:** Manuel Göbel, Martin Gschnell

**Affiliations:** Klinik für Dermatologie und Allergologie, Universitätsklinikum Gießen und Marburg GmbH, Standort Marburg, Baldingerstr., 35043 Marburg, Deutschland

## Anamnese

Eine 30-jährige Frau stellte sich mit seit mehreren Wochen bestehenden, initial juckenden und nachfolgend stechend brennenden Hautveränderungen in Form von erythematösen Makeln und Papeln in unserer Hochschulambulanz vor. Der Befund habe sich initial vom rechten Handrücken über den gesamten Arm und anschließend die übrigen Extremitäten ausgebreitet. Weitere Beschwerden, insbesondere Fieber oder Schüttelfrost, wurden verneint. Zeitlich seien dem Auftreten erster Effloreszenzen ein Zeckenbiss am Bein sowie ein Kratzer durch die ausschließlich in der Wohnung lebende Hauskatze am Unterarm vorausgegangen. Chronische Erkrankungen, Allergien oder regelmäßige Medikamenteneinnahme wurden verneint.

## Untersuchung

**Figure 1:**
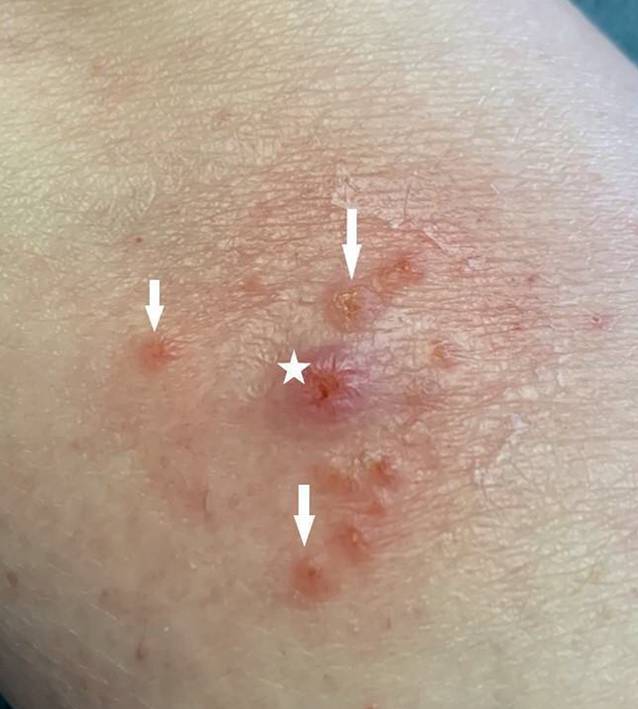

**Figure 2:**
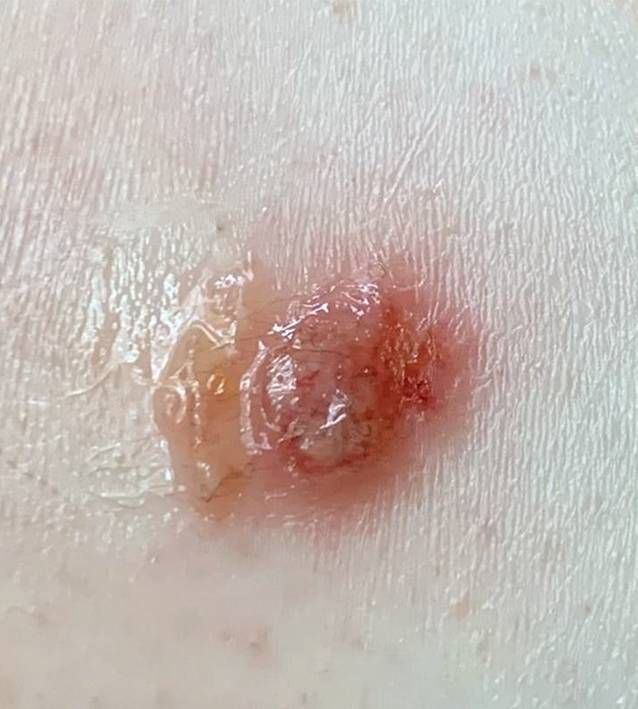

**Figure 3:**
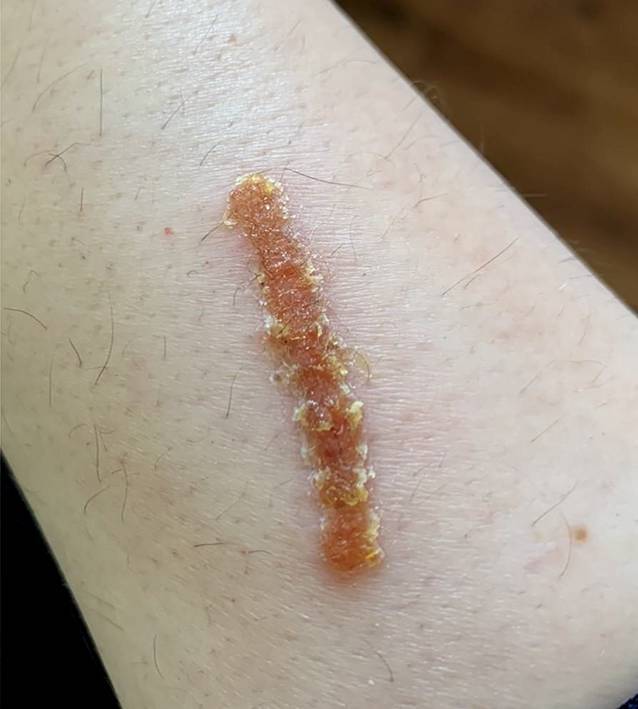

Die Patientin präsentierte sich in altersentsprechend gutem Allgemein- und normalem Ernährungszustand. Es zeigten sich an den streckseitigen Extremitäten mit Betonung des linken Unterarms multiple Papeln (vgl. Abb. 1, Pfeil) und Noduli (vgl. Abb. 1, Sternchen und Abb. 2) auf erythematösem Grund, am rechten Unterarm zu teils linienartig hyperkeratotischen Plaques konfluierend (vgl. Abb. 3), sowie vereinzelt erythematöse Makeln und Kratzexkoriationen. Kopfhaut, Gesicht und Mundschleimhaut waren erscheinungsfrei. Eine (generalisierte) Lymphadenopathie lag nicht vor.

## Diagnostik

Laborchemisch zeigten sich das Differenzialblutbild unauffällig und das C‑reaktive Protein normwertig. Antinukleäre Antikörper zeigten sich normtitrig. Antikörper gegen Gewebstransglutaminase ließen sich nicht nachweisen. Histologisch ergab sich aus einer läsional entnommenen 4-mm-Stanzbiopsie eines Nodulus am linken Unterarm das Bild eines subakuten Ekzems mit Eosinophilie. In der direkten Immunfluoreszenz hingegen zeigte sich kein pathologischer Befund. Pathogene Bakterien, Pilze und Herpesviren (Herpes simplex Typ I und II) ließen sich in multiplen Abstrichen von der betroffenen Haut nicht nachweisen. Eine Infektion mit *Borrelia burgdorferi* ließ sich serologisch ausschließen. Auffällig zeigten sich hingegen erhöhte IgM- bei gleichzeitig fehlenden IgG-Antikörpern gegen *Bartonella henselae*.

## Therapie und Verlauf

**Figure 4:**
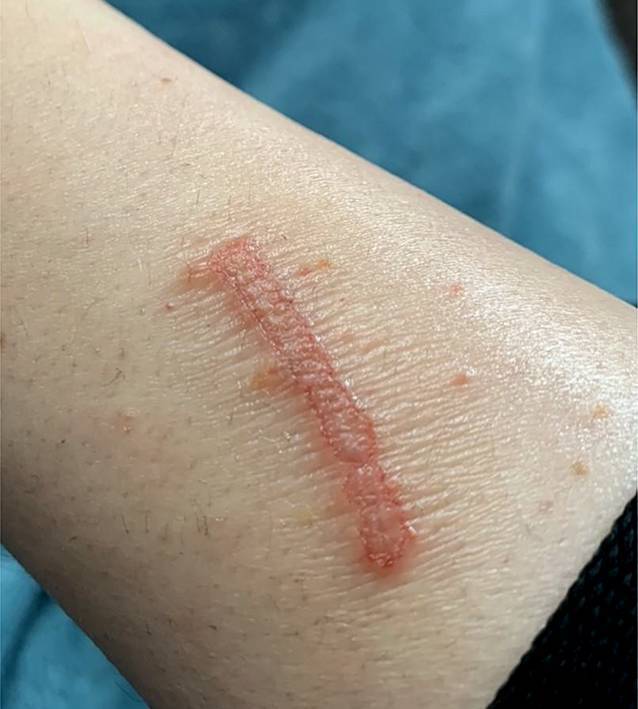

**Figure 5:**
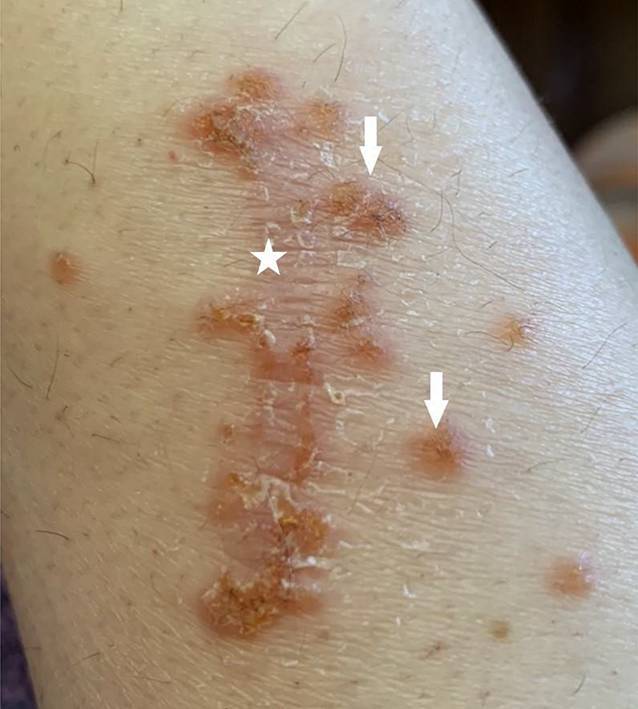

Unter dem initialen klinischen Verdacht eines pruriginösen Ekzems behandelten wir nach vorsichtigem Entfernen der Hyperkeratosen (vgl. Abb. 4) zunächst topisch mit einer Kombination aus Betamethasonvalerat und Fusidinsäure, worunter sich eine deutliche Linderung des Pruritus und ein allmähliches Abblassen des Lokalbefundes (vgl. Abb. 5) zeigten. Nach Erhalt der zuvor genannten Untersuchungsergebnisse bei gleichzeitig gutem klinischem Ansprechen auf die verordnete Lokaltherapie entschlossen wir uns für die Fortführung der topischen Therapie mit Fusidinsäure. Es kam zu einer vollständigen Remission des klinischen Befundes. In der später durchgeführten serologischen Kontrolle hinsichtlich *Bartonella henselae* zeigte sich eine Serokonversion von IgM zu IgG, was für eine therapierte bzw. durchgemachte Infektion sprach.

## Diskussion

Der serologische Nachweis von IgM-Antikörpern gegen *Bartonella henselae* in Verbindung mit dem passenden klinischen Befund, bestehend aus linienartig angeordneten pruriginösen Papeln und Noduli auf erythematösem Grund nach anamnestisch vorausgegangenem Kratzer durch die Hauskatze am betroffenen Unterarm, führten uns zur Diagnose einer Katzenkratzkrankheit.

Dabei handelt es sich um eine Infektion mit dem gramnegativen aeroben Stäbchenbakterium *Bartonella henselae*. Es sind 3 humanpathogene Spezies bekannt: *B. quintana* (Erreger des Fünftagefiebers), *B. bacilliformis* (Erreger des Oroya-Fiebers und der peruanischen Warzen) sowie *B. henselae* (Erreger der Katzenkratzkrankheit und der bazillären Angiomatose). Vektoren sind neben der Sandfliege der Kot des Katzenflohs; es handelt sich um eine Zoonose. Die Inkubationszeit beträgt in der Regel zwischen 7 und 10 Tagen. Angaben zur Prävalenz für Deutschland schwanken je nach literarischen Angaben zwischen 1 und 9 pro 100.000 Einwohner [1]. Die befallenen Katzen zeigen sich in der Mehrzahl der Fälle klinisch inapparent [2]. Zusätzlich zum kutanen Befund kann es bei der Katzenkratzkrankheit beim Menschen zu passageren (sub)febrilen Temperaturen [3] und bei immunsupprimierten Patienten zu septischen oder enzephalischen [4] Verläufen sowie zur bazillären Angiomatose [5] mit unbehandelt häufig letalem Verlauf kommen. Letztere stellt eine HIV/AIDS-assoziierte Erkrankung dar und präsentiert sich klinisch in Form von (disseminiert) über das Integument verteilten erythematösen Knötchen bis hin zu exulzerierten Knoten, die nicht selten einem Granuloma pyogenicum ähneln können. Ursächlich ist die vermehrte Freisetzung von VEGF („vascular endothelial growth factor“), die zu einer Proliferation der Endothelzellen führt. Nicht selten zeigt sich der klinische Verlauf protrahiert mit fieberhafter Begleitsymptomatik. Ein Befall innerer Organe wie Knochenmark, Milz und Leber ist möglich, wobei Letztere zystisch zersetzt wird (Peliosis hepatis), was mit einem letalen Verlauf vergesellschaftet sein kann. Bei der Mehrheit der von der Katzenkratzkrankheit betroffenen Patienten zeigt sich eine lokalisierte Lymphadenopathie [3]. Bei Kindern und Jugendlichen ist die Erkrankung die häufigste Ursache einer benignen regionalen Lymphadenopathie in dieser Altersklasse. Pneumonie und Endokarditis stellen weitere gefürchtete, wenn auch seltene Komplikationen dar. Trotz hoher Spontanheilungsrate kann die Lymphadenopathie unter Umständen über Monate persistieren.

*Bartonella henselae* kann mittels kultureller Anzucht (Goldstandard), PCR oder serologischen Antikörpertests nachgewiesen werden. Therapie der Wahl ist neben suppurativen und symptomatischen Maßnahmen die systemische Behandlung mit Makroliden und Tetrazyklinen [6] sowie Rifampicin, Trimethoprim-Sulfamethoxazol und Ciprofloxacin [7], sofern erforderlich. Spontanheilungen werden jedoch gerade bei immunkompetenten Patienten häufig beobachtet. Ein Impfstoff existiert nicht. Gesunde Trägerkatzen sollten antibiotisch behandelt werden, wenn diese mit immunsupprimierten Menschen zusammen leben. Ebenso sollten klinisch erkrankte Katzen (beispielsweise im Falle einer Bartonellen-verursachten Uveitis oder Endokarditis) antibiotisch behandelt werden. Prophylaktisch sollte wildes Spielen mit Katzen vermieden werden sowie deren Krallen gekürzt und das Fell regelmäßig auf Flohbefall untersucht werden.

## Fazit für die Praxis

- Bartonellen sind gramnegative aerobe Stäbchenbakterien.
- Drei humanpathogene Arten sind bekannt: *B. quintana, B. bacilliformis, B. henselae*.
- *B. henselae* ist Erreger der Katzenkratzkrankheit sowie der bazillären Angiomatose und wird über den Kot des Katzenflohs übertragen.
- Betroffene Katzen sind meist asymptomatisch; in seltenen Fällen können Uveitis und Endokarditis auftreten.
- Juckende Papeln und Noduli, (sub)febrile Temperaturen und Lymphadenopathie sind die häufigsten klinischen Symptome beim Menschen.
- Zum Nachweis eignen sich kulturelle Anzucht (Goldstandard), PCR und Antikörperbestimmung im Serum.
- Therapeutisch kommen u. a. Makrolide und Tetrazykline zum Einsatz; Spontanheilungen sind häufig.
- Ein Impfstoff existiert nicht.
